# Multisensory and lexical information in speech perception

**DOI:** 10.3389/fnhum.2023.1331129

**Published:** 2024-01-08

**Authors:** Josh Dorsi, Simon Lacey, K. Sathian

**Affiliations:** ^1^Department of Neurology, Penn State College of Medicine, Hershey, PA, United States; ^2^Department of Neural and Behavioral Sciences, Penn State College of Medicine, Hershey, PA, United States; ^3^Department of Psychology, Penn State Colleges of Medicine and Liberal Arts, Hershey, PA, United States

**Keywords:** multisensory, lexical, speech perception, sound symbolism, audiovisual

## Abstract

Both multisensory and lexical information are known to influence the perception of speech. However, an open question remains: is either source more fundamental to perceiving speech? In this perspective, we review the literature and argue that multisensory information plays a more fundamental role in speech perception than lexical information. Three sets of findings support this conclusion: first, reaction times and electroencephalographic signal latencies indicate that the effects of multisensory information on speech processing seem to occur earlier than the effects of lexical information. Second, non-auditory sensory input influences the perception of features that differentiate phonetic categories; thus, multisensory information determines what lexical information is ultimately processed. Finally, there is evidence that multisensory information helps form some lexical information as part of a phenomenon known as sound symbolism. These findings support a framework of speech perception that, while acknowledging the influential roles of both multisensory and lexical information, holds that multisensory information is more fundamental to the process.

## Introduction

Both lexical and multisensory information are known to support the perception of speech. For example, it is easier to identify speech comprising words rather than non-words (Hirsh et al., 1954) and speech conveyed through multiple rather than single sensory channels (i.e., audiovisual speech vs. auditory-only speech; Sumby and Pollack, 1954). In natural settings, speech typically involves real words (providing lexical information) spoken by talkers we can both see and hear (providing multisensory information). Thus, understanding how multisensory and lexical information are processed in relation to each other is important for a comprehensive understanding of the speech mechanism. Although work jointly testing lexical and multisensory information in speech perception is limited, some inferences can be made from such studies, as well as from work examining multisensory and lexical effects separately. Based on a review of this literature, we argue in this perspective that multisensory information plays a more fundamental role in speech perception than lexical information.

The McGurk effect is one of the most prominent demonstrations that lexical and multisensory information interact during speech perception. It refers to the finding that for some audiovisually incongruent speech, listeners hear the visual, not the auditory, signal (i.e., auditory /ba/ + visual /da/ is heard as /da/; McGurk and MacDonald, 1976). While originally studied at the level of syllables, the effect is influenced by the lexical composition of the incongruent speech, with visually consistent percepts being more common for auditory-non-word + visual-word (e.g., auditory /bεf/ + visual /dεf/) compared to auditory-word + visual-non-word (e.g., auditory /bænd/ + visual /dænd/; Brancazio, 2004). Such findings demonstrate the interactive effects of lexical and multisensory information in speech perception but do not allow us to distinguish whether (a) lexical information changes audiovisual integration or (b) lexical information influences some post-integration process.

Understanding the relative roles of lexical and multisensory processing in speech perception is vital for a fuller understanding of language comprehension. There is a lack of consensus on this issue, with some researchers favoring a more fundamental role of multisensory information (Rosenblum, 2008), whereas others assume the primacy of lexical information (Ostrand et al., 2016). Here, we review evidence that multisensory processing precedes lexical processing, affects the perception of pre-lexical speech units, and influences the formation of lexical representations. These lines of evidence support the contention that multisensory information is more fundamental to speech perception than lexical information.

## What is the relative timing of lexical and multisensory processing?

Information processed earlier can influence information processed later. Both lexical and multisensory processing can occur rapidly (e.g., Baart and Samuel, 2015). This section examines studies that include multisensory and lexical information within the same paradigm to evaluate whether one might be handled earlier.

Baart and Samuel (2015) measured event-related potentials (ERPs) in response to three-syllable words and pseudowords presented auditorily, visually, or audiovisually. ERPs relative to the onset of the third syllable, where the word-determining information occurred, showed significant effects of multisensory information at earlier time windows (0–50 ms) than for lexical information (100–150 ms). An analogous second experiment also found that multisensory effects (50–100 ms) were earlier than lexical effects (150–200 ms). The multisensory effects do not seem to be driven by the early availability of visual information, as they were associated with frontal electrodes. These data have notably high subject-level variability (see Baart, 2016); however, they resemble results obtained in another multisensory-lexical ERP study (Basirat et al., 2018) investigating the word repetition effect (the finding of facilitated processing for repeated words). Basirat et al. (2018) measured ERPs starting at the onset of initially presented and repeated auditory-only and audiovisual words. They found that the earliest effect of lexical information (word repetition) was in the 170–280 ms window; in contrast, modality had a main effect in the 0–80 ms window, suggesting that multisensory processing preceded lexical processing (Basirat et al., 2018).

Ostrand et al. (2016) investigated the relative timing of lexical and multisensory processing by testing whether semantic priming, a lexical process, is sensitive to multisensory integration. Auditory-only target words (e.g., /wɜrm/) were categorized faster when they followed audiovisual-incongruent prime words with an auditory component semantically related to the target word (i.e., auditory /bet/ + visual /det/); semantic priming was consistent with the auditory channel of incongruent primes. These incongruent primes could be integrated such that participants “heard” either the visual or the auditory word (Brancazio, 2004). Dorsi et al. (2023) replicated the semantic priming paradigm of Ostrand et al. (2016) and included a McGurk effect assessment for the primes. This study found that priming to the auditory words corresponded to how likely the incongruent stimulus was to be heard as the auditory word. Likewise, primes frequently heard as the visual word were associated with priming consistent with the visual word (Dorsi et al., 2023). This suggests that multisensory integration precedes lexical processing because semantic priming appears contingent on the multisensory interactions determining the incongruent word’s perception. However, alternative explanations also exist, such as the possibility of a lexical contribution to the perception of the incongruent word. Using ERPs in a semantic priming paradigm might be helpful to confirm that multisensory perception, indexed by the McGurk effect, precedes the availability of lexical information.

Baart and Samuel (2015) and Basirat et al. (2018) measured multisensory and lexical effects in a time-sensitive way. Both studies were interested in the P2, an ERP whose latency and amplitude are modulated approximately 200 ms after relevant lexical or multisensory information appears in the speech signal (see Baart and Samuel, 2015 for a discussion). The P2 is assumed to be associated with early lexical processes (Basirat et al., 2018), and indeed, both studies found lexical effects in the P2 time window. However, both studies also converge in showing effects of multisensory information in earlier windows (e.g., 0–80 ms) than those for lexical information. The results of Dorsi et al. (2023) are consistent with this conclusion since lexical processing apparently depends on the outcome of multisensory integration, although this question should be more thoroughly tested to exclude alternative possibilities.

## Does multisensory information influence what lexical information is processed?

While lexical processing might begin with pre-lexical speech units, there is evidence that multisensory information shapes the perception of even the most basic pre-lexical information. For example, visual speech affects the perception of pre-phonetic auditory information such as voice-onset-time (VOT), the time from acoustic onset to the sudden increase in acoustic energy (Green and Miller, 1985) that distinguishes phonemes such as /b/ from /p/ (/b/ = shorter VOT). Despite its link to the acoustic signal, VOT is perceived as being shorter when accompanied by fast vs. slow visual speech (Green and Miller, 1985), suggesting that multisensory interactions influence a pre-lexical feature that presumably is involved with initial lexical processing.

Multisensory input also influences the perception of the speech signal’s more basic acoustic parameters (e.g., Plass et al., 2020). For example, the visible shape of the mouth opening improves the perception of degraded auditory speech through its influence on the perception of spectro-temporal properties of the acoustic signal (e.g., formants; Plass et al., 2020). Likewise, the correlation between visible changes in the area of the mouth opening and the auditory speech envelope corresponds to audiovisual improvement of speech-in-noise perception (Grant and Seitz, 2000). Activity in auditory cortical areas is known to correlate with the auditory speech envelope (e.g., Abrams et al., 2008); the addition of visual speech (Crosse et al., 2015) or even vibrotactile speech (Riecke et al., 2019) improves this cortical tracking. Likewise, audiovisual speech influences auditory cortical activity (Okada et al., 2013). Moreover, visual speech influences auditory speech-associated activity in the brainstem (Musacchia et al., 2006) and the cochlea (Namasivayam et al., 2015). While these latter effects may result from feedback from cortical locations, they demonstrate how multisensory input influences the neural fate of even the most basic auditory information. Thus, the perception and neural handling of basic speech information, even with lexical feedback (e.g., Marian et al., 2018; Li et al., 2020), is not free from multisensory influences.

## Is lexical information formed independent of multisensory processing?

It takes months of experience before lexical information becomes useful to listeners (e.g., Jusczyk et al., 1994). Multisensory effects on speech perception likely occur while lexical representations are being formed in childhood (Walton and Bower, 1993). A set of findings related to sound symbolism is consistent with this notion. Sound symbolism is the association between the sound of a word and its meaning. While sound symbolism is still poorly understood, we review evidence here suggesting that it may be inherent to language processing, involve multisensory processing, and support language acquisition. These points suggest the intriguing possibility that multisensory information is involved in forming some lexical information.

Sound symbolism may be inherent to language. The sound-symbolic associations of pseudowords (e.g., /buba/ sounds rounded, /kiki/ sounds pointed: Ramachandran and Hubbard, 2001) generalize to phonetic-to-meaning correspondences in real words (Sidhu et al., 2021). This correspondence is common across the world’s languages (Blasi et al., 2016). Sound symbolism also seems to be related to the neural basis of language. In a recent functional magnetic resonance imaging (fMRI) study, a multivoxel pattern analysis (MVPA) indicated that activity in language-associated areas such as the left supramarginal gyrus and Broca’s area in the left inferior frontal gyrus could distinguish rounded/pointed stimuli more accurately for sound symbolically matched pseudoword-shape pairs (e.g., /molo/ + rounded shape) than for mismatched pairs (e.g., /molo/ + pointed shape) (Barany et al., 2023).

There is also evidence that sound symbolism may involve multisensory processing. For example, sound symbolically matched audiovisual pseudoword-shape pairs produce more activation in auditory areas than unmatched pairs (Barany et al., 2023). In visual areas, the activation difference between mismatched and matched pseudoword-shape pairs correlates with behavioral measures of implicit pseudoword-shape associations (Peiffer-Smadja and Cohen, 2019). Likewise, MVPA indicates that activity in early visual areas more accurately distinguishes rounded and pointed stimuli that are part of sound symbolically matched, as opposed to mismatched, pseudoword-shape pairs (Barany et al., 2023). While the exact nature of the neural computations underlying sound symbolism are still not understood, these findings indicate that sound symbolism may, at least partly, involve multisensory processing.

Moreover, sound symbolism may support language acquisition. The sound symbolism bootstrapping hypothesis proposes that sound-symbolic associations facilitate initial word learning (Imai and Kita, 2014). Indeed, words rated as sounding like their meaning are overrepresented in the earliest words learned by children (Perry et al., 2017). Infants are sensitive to sound-symbolic correspondences; four-month-olds prefer sound symbolically matched to mismatched speech-shape pairs (Ozturk et al., 2013), and 14-month-olds are better at learning sound symbolically matched than mismatched labels for novel shapes (Imai et al., 2015). Adults are better at learning sound symbolically congruent than incongruent pseudoword-shape mappings (Revill et al., 2018), are more accurate in learning the correct than incorrect meanings of sound-symbolic foreign language words (Lockwood et al., 2016), and are better than chance at choosing the correct meaning of sound-symbolic foreign word pairs (Revill et al., 2014). The role of multisensory interactions in sound symbolism suggests that, at least for some words, multisensory processes influence the formation of lexical representations.

## Discussion and conclusion

In this perspective, we reviewed literature suggesting that multisensory information is more fundamental to speech perception than lexical information. Three sets of observations support our argument: there may be earlier processing of multisensory information; the basic units of lexical representations are sensitive to multisensory information; and, through sound symbolism, some lexical representations may be formed with multisensory inputs. Each of these ideas requires further testing. Such testing could include methods with high temporal resolution to simultaneously measure the timing of multisensory processes in relation to the recovery of pre-lexical information (e.g., phonetic features or spectro-temporal acoustic parameters) and subsequent lexical processes. Experiments that more directly test the role of multisensory interactions in sound symbolism and examine the lexical effects of sound symbolism will also be useful. While work remains to be done, we conclude that multisensory information is likely more fundamental to speech perception than lexical information. There are clinical implications of this view. For example, while cochlear implant recipients demonstrate reduced multisensory integration, multisensory information reliably supports word recovery in this population (Stevenson et al., 2017). Recent work has also found word perception improvements when cochlear implant recipients wore a device that transduced the acoustic speech signal into vibratory stimulation in real time (Fletcher et al., 2019). Similarly, people with aphasia show improved lexical processing in challenging listening conditions when speech is presented in a multisensory context (Krason et al., 2023). These observations demonstrate the importance of considering the relative impacts of multisensory and lexical information on speech processing, as the present perspective has discussed.

## Data availability statement

The original contributions presented in this study are included in this article/supplementary material, further inquiries can be directed to the corresponding author.

## Author contributions

JD: Conceptualization, Writing – original draft, Writing – review & editing. SL: Writing – review & editing. KS: Writing – review & editing.
